# On the Etiology of Recurring Facial Neuralgia after Neurectomy of One of the Affected Branches of the Fifth Nerve

**Published:** 1890-01

**Authors:** A. Obalinski

**Affiliations:** Cracow


### Abstracts.

### ON THE ETIOLOGY OF RECURRING FACIAL NEURALGIA AFTER NEURECTOMY OF ONE OF THE AFFECTED BRANCHES OF THE FIFTH NERVE.

By A. OBALINSKI, in Cracow.

Abstract from *Wiener Klin. Wochensch., No. 41, 1889.*

INASMUCH as the results of neurectomy, especially of the branches of the fifth nerve, are so uncertain, and not lasting, the applied methods of operation so little established, and the action of the parts of the body governed by these nerves still so varied, the author believes that every article, throwing light upon this obscure subject, is welcome, and therefore submits the report of the following case:

Joseph Szewczky, peasant, about 62 years old, came under treatment in January, 1881, for an intolerable facial neuralgia, the result of the kick of a horse, twenty years previously. In the beginning, the right cheek and upper lip were affected, and the trouble intensified by sensitive scars. Latterly, the attacks came so often and severely that the patient was desirous of undergoing anything for relief.

On January 26, 1881, neurectomy (*a la* Wagner) of the right infraorbital nerve was made, and a piece, one centimeter long, removed from the canal. In one month, the patient was discharged, free from neuralgic attacks during this time, and the wound entirely healed, with scarcely perceptible scar.

On October 23, 1882, twenty-one months after operation, he returned, saying that for one full year he had been free from pain, but that, since this time, the attacks were becoming more severe and often each month.

On July 2, 1883, two and one-half years after the operation, he came again to the Surgical clinic, complaining of very intense neuralgic attacks, which were quite as severe as before operation. The scar was opened, and the thermo-cautery applied to the nerve stump. Relief lasted only a very short time, and soon the attacks reached their former intensity. Mikulicz, the colleague of Obalinski, then resected the remainder of the nerve, up to the foramen rotundum, by means of temporary resection of the superior maxilla (Langenbeck's method). The wound healed *per primam*, and the pain was completely abolished.

This condition, likewise, lasted one year, when the pain began to slowly reappear, and in the third year after this operation had reached the previous degree of intensity. In the Summer of 1887, Docent Dr. Trzebicky operated after the method of Lücke-Braun-Lossen, the coronoid process having been resected, to better reach the fissure. Soon the pain reappeared in its greatest intensity, with the additional misfortune of anchylosis of the right inferior maxillary articulation, so that the patient could scarcely open his mouth and receive sufficient nourishment. (This is the second case in which the author has seen anchylosis occur, and which, in his opinion, speaks strongly against this method of operating.)

In June, 1889, two years after the last, and eight and one-half years after the first operation, the patient, tormented with frightful attacks, came again to Obalinski, whose examination showed the present condition to be as follows: Pain is limited to the region of the right cheek and the right half of the upper lip, is started up by pressure over the right infraorbital foramen and by touching the upper lip on the right side, and is so great that the patient is willing to undergo the most serious operation for the sake of relief. Pressure over the inferior maxilla, and over the right superior border of the orbit, brings on no attacks of pain. The right superior maxillary region shows a complete system of young scars, which unite with older ones on the upper lip. The lower jaw was immovable, on which account the patient was nourished with fluids, although small pieces of solid food could pass on the left side into the mouth, through the place of an absent tooth.

Guided by previous experience, the author determined to open up the foramen rotundum, remove its bony edge, and extirpate the remaining part of the second branch, up to the Gasserian ganglion, and to do this by temporarily resecting the superior maxilla, removing some of the zygoma at the same time, to gain more room.

On June 6, 1889, Obalinski proceeded to operate after the method of Langenbeck, but was compelled to give it up, because the maxilla could not be raised, owing, probably, to callous formation resulting from the previous similar operation. He, therefore, removed the anchylosis of the lower jaw, by a method previously described (*Wiener Med. Presse*, Nr. 9, 1889). In the beginning, he had intended to leave the joint in place, but thinking that, from the nature of wound cavity left, copious suppuration and necrosis of the joint might occur, he removed the same, together with a piece of the nerve several centimeters long, making a larger but more manageable wound.

The lower jaw could be depressed, and the mouth sufficiently opened, and just in time, for the blood from the incompleted resection of the superior maxilla began to collect in the throat, and threaten the patient with suffocation.

The following morning, the patient was completely free from pain, the wound healed pretty rapidly, and the attacks did not recur during the month following, after which the patient was discharged.

The author does not accept the explanation that the pain was caused in consequence of the reflex effect of a large operation, because two years previously an equally large operation did not bring relief, and at any rate such influence would only last for a very short time and not act so radically as in the case under consideration.

Thorough consideration leads the author to explain the fact by the anastomoses between the terminal branches of the second, and those of the nerves lying in the immediate neighborhood. He instances as analogous the case of Nélaton where extirpation of the median nerve did not affect the innervation of the parts supplied by this nerve; also the similar observations of Langier, Richet, Kraussold, Küster and Gzeparowicz, which are explained partly by Letievant's theory of "Motilite Supplee," and, especially in the sensory regions, by anastomoses between the terminal branches of the three great nerve trunks of the upper extremity. (Broca, Richet.)

Beside Nélaton's case, the author can find no other similar case affecting other nerves recorded; notwithstanding that no other regions of the body appears more adapted than that of the face where the entire plexus of the nerve lies by that of another, and where many ganglia connect with the fibers springing from various nerve trunks.

The text-books of anatomy teach us nothing of the direct anastomosis between the second and third branches of the fifth nerve; we know only of an indirect communication between them through the facial nerve; still the above quoted case seems to prove the existence of direct anastomosis. Upon no other supposition can the author explain why the pain should have remained away when the third branch only was resected, the second not having been touched. It must, therefore, be believed that direct communication between both nerves in consequence of previous neurectomies was established, and which, notwithstanding the destroyed conduction in the path of the branches of the second nerve, maintained the terminal fibers of the same, and so contributed to the origin of the recurring attacks.

The article is concluded with the remark that—

> Although this observation at the present time stands alone, it is still capable of throwing some light upon the recurrence of facial neuralgia, and, perhaps, of recommending neurectomy of the neighboring nerve for methodical trial in severe recurring cases.

